# Draft Genome Sequence of *Deefgea* sp. Strain CFH1-16, Isolated from the Intestine of the Freshwater Fish Hypomesus nipponensis

**DOI:** 10.1128/MRA.01389-20

**Published:** 2021-05-13

**Authors:** Ji-Hye Han

**Affiliations:** a Microbial Research Department, Nakdonggang National Institute of Biological Resources, Sangju-si, Republic of Korea; Portland State University

## Abstract

Reported here is the draft genome sequence of *Deefgea* sp. strain CFH1-16, isolated from the intestine of a Hypomesus nipponensis fish collected from the Republic of Korea. The genome sequence was assembled into two contigs with 48.2% G+C content. These genome data could improve our understanding of adaptation in the fish intestine.

## Abstract (precis)

We report the draft genome sequence of *Deefgea* sp. strain CFH1-16, isolated from the intestine of a Hypomesus nipponensis collected from the Republic of Korea. The genome sequence was assembled into two contigs with 48.2% G+C content. This genome data could improve our understanding of adaptation in the fish intestine.

## ANNOUNCEMENT

Eukaryotic hosts are important habitats for microbes, each host having a unique and intimate interaction with its microbiomes. The intestines are particularly important, and those of aquatic animals harbor especially denser bacterial communities, as water is an ideal medium for bacterial growth (1).

Here, we collected wild Hypomesus nipponensis from a freshwater lake in Chuncheon, Republic of Korea (38°2′21.3″N, 127°38′55.31″). This species is a well-known food fish native to the lake and has the unique characteristics of growing optimally at low temperature and spawning from the winter to early spring (2). After homogenization of intestine samples with a tissue grinder, these diluted samples were incubated on Reasoner's 2A (R2A) agar plates at 20°C for 2 weeks with oxygen, and fish intestine bacteria were isolated and taxonomically identified via the EzBioCloud server (3) using the amplified 16S rRNA gene sequences (4); among the organisms identified were strains of the genus *Deefgea*.

The genus *Deefgea*, first reported by Stackebrandt (5), contains poly-β-hydroxybutyrate granules. The strain CFH1-16 has the same characteristics as *Deefgea* spp. but is able to grow at low temperature (under 4°C). In the present study, the genome sequence of the strain CFH1-16, the first host-associated *Deefgea* species identified, is reported.

Strain CFH1-16 was grown aerobically on R2A agar plates for 3 days at 25°C. Genomic DNA was extracted and purified using the DNeasy blood and tissue kit (Qiagen) and the Wizard genomic DNA purification kit (Promega), respectively. After DNA shearing with g-TUBE (Covaris), a 20-kb SMRTbell genome library was prepared using the SMRTbell template prep kit 1.0 (Pacific Biosciences) with genomic DNA in the optimal size range (for 3 to 10 kb). Sequencing was performed using the PacBio RS II single-molecule real-time (SMRT) sequencing platform according to the manufacturer’s instructions at DNA Link (Seoul, Republic of Korea) and produced 13,699 bp of long reads and 591,401,273 bp after subread filtering with the default parameters defined by Pacific Biosciences. The bacterial genome *de novo* assembly was carried out with the Hierarchical Genome Assembly Process (HGAP) v3.0 (6). The draft genome sequence of strain CFH1-16 was submitted to NCBI for automated annotation using the Prokaryotic Genome Annotation Pipeline (PGAP). We searched for potential coding sequences using the Basic Local Alignment Search Tool (BLAST) against the UniProt (7), Pfam (8), and Clusters of Orthologous Groups of proteins (COG) (9) databases. CRISPRCasFinder v4.2.20 (10) was utilized for CRISPR/Cas analysis, and rRNA and tRNA features from the assembled contigs were predicted using RNAmmer v1.2 (11) and tRNAscan-SE v1.21 (12), respectively. Additionally, the average nucleotide identity (ANI) value with closely related species was determined using the Orthologous Average Nucleotide Identity Tool (OAT) software (13).

The genome sequence was assembled into two contigs with an *N*_50_ value of 3,401,602 bp and a total genome size of 3,451,982 bp with 48.2% G+C content, 4,206 coding DNA sequences (CDSs), 24 rRNAs, and 81 tRNAs. Four CRISPR sequences and a CAS cluster (CAS-TypeIF) were identified using CRISPRCasFinder v4.2.20. The genome contains CDSs similar to genes for cold shock protein, poly-β-hydroxybutyrate polymerase, and several metal resistance proteins. The ANI values (below 79.37%) between strain CFH1-16 and the closely related species Deefgea rivuli (GenBank accession number JHVM01000000) and Deefgea chitinilytica (WOFE00000000) demonstrated that strain CFH1-16 may represent a novel species of the genus *Deefgea*.

### Data availability.

All data are available in the NCBI under BioProject accession number PRJNA590067. The raw sequencing reads have been deposited in the SRA under accession number SRR12664465. The assembled genome has been deposited under accession number WLYT00000000.1.
